# An evolution strategy approach for the balanced minimum evolution problem

**DOI:** 10.1093/bioinformatics/btad660

**Published:** 2023-10-27

**Authors:** Andrea Gasparin, Federico Julian Camerota Verdù, Daniele Catanzaro, Lorenzo Castelli

**Affiliations:** Dipartimento di Ingegneria e Architettura, Università degli Studi di Trieste, Trieste 34127, Italy; Dipartimento di Matematica e Geoscienze, Università degli Studi di Trieste, Trieste 34128, Italy; Center for Operations Research and Econometrics (CORE), Université Catholique de Louvain, Louvain-la-Neuve 1348, Belgium; Dipartimento di Ingegneria e Architettura, Università degli Studi di Trieste, Trieste 34127, Italy

## Abstract

**Motivation:**

The *Balanced Minimum Evolution* (BME) is a powerful distance based phylogenetic estimation model introduced by Desper and Gascuel and nowadays implemented in popular tools for phylogenetic analyses. It was proven to be computationally less demanding than more sophisticated estimation methods, e.g. maximum likelihood or Bayesian inference while preserving the statistical consistency and the ability to run with almost any kind of data for which a dissimilarity measure is available. BME can be stated in terms of a nonlinear non-convex combinatorial optimization problem, usually referred to as the *Balanced Minimum Evolution Problem* (BMEP). Currently, the state-of-the-art among approximate methods for the BMEP is represented by FastME (version 2.0), a software which implements several deterministic phylogenetic construction heuristics combined with a local search on specific neighbourhoods derived by classical topological tree rearrangements. These combinations, however, may not guarantee convergence to close-to-optimal solutions to the problem due to the lack of solution space exploration, a phenomenon which is exacerbated when tackling molecular datasets characterized by a large number of taxa.

**Results:**

To overcome such convergence issues, in this article, we propose a novel metaheuristic, named PhyloES, which exploits the combination of an exploration phase based on Evolution Strategies, a special type of evolutionary algorithm, with a refinement phase based on two local search algorithms. Extensive computational experiments show that PhyloES consistently outperforms FastME, especially when tackling larger datasets, providing solutions characterized by a shorter tree length but also significantly different from the topological perspective.

**Availability and implementation:**

The software and the data are available at https://github.com/andygaspar/PHYLOES.

## 1 Introduction

Distance methods constitute a well-consolidated theoretical and algorithmic framework to carry out practical phylogenetic analyses. These methods are typically based on hypotheses and assumptions that are considerably simpler than those at the core of more sophisticated estimation methods, such as *Maximum Likelihood* (ML) or *Bayesian Inference* (BI), and this fact can make them poor at modelling complex evolutionary processes (Schwartz 2019, Catanzaro *et al.* 2022). However, off-the-shelf distance methods can run with almost any kind of data for which a dissimilarity measure is available and are computationally less demanding than more sophisticated estimation methods based on ML and BI. Moreover, several estimation models in distance methods, including *Minimum Evolution under Ordinary Least Squares* (Gascuel 2005), *Balanced Minimum Evolution* (Desper and Gascuel 2002), or *Minimum Evolution under Linear Programming* (Catanzaro *et al.* 2015), share with ML and BI the highly desirable property of being statistically consistent, i.e. as long as the estimated evolutionary distances between the molecular sequences are unbiased estimations of their true evolutionary distances, the optimal phylogenies to these estimation models approach the true phylogeny for a larger and larger number of sites analysed. One statistically consistent phylogenetic estimation model that arises from distance methods and that is central to this article is Balanced Minimum Evolution (Gascuel 2005). This model can be stated in terms of a discrete non-linear non-convex optimization problem defined over unrooted binary trees (Catanzaro *et al.* 2012). Specifically, consider a set X={1,2,…,n} of n≥3 distinct aligned molecular sequences (such as DNA, RNA, or codon sequences), hereafter referred to as *taxa*, and a n×n symmetric distance matrix D, whose generic entry $d_{ij}$ represents a measure of the dissimilarity between the pair of taxa i,j∈X. Then, the *Balanced Minimum Evolution Problem* (BMEP) consists in finding a *phylogeny T* of *X* (i.e. an unrooted binary tree *T* having *X* as a leaf set) that minimizes the following *length function*


(1) $$L(T)=\sum_{i\in X}\sum_{j\in X\setminus \{i\}}\frac{d_{ij}}{2^{\tau_{ij}-1}},$$

where $\tau_{ij}$ represents the *path-length* between taxa *i* and *j* in *T*, i.e. the number of edges belonging to the (unique) path in *T* connecting taxon *i* to taxon *j* (Pardi 2009, Catanzaro *et al.* 2012).

The BMEP, recently reviewed in Catanzaro *et al.* (2022), is NP-hard and inapproximable within a $c^{n}$-factor, for some positive constant c>1, unless P=NP (Fiorini and Joret 2012). The BMEP is instead polynomially solvable if the input distance matrix D is *additive* (Gascuel 2005), i.e. if its entries satisfy


(2) $$d_{ij}+d_{kr}\leq \max\{d_{ik}+d_{jr},d_{ir}+d_{jk}\}\;\forall \;i,j,k,r\in X.$$

If the input distance matrix D is just *metric*, i.e. if its entries satisfy the triangle inequality, then the optimal solution to the BMEP can be approximated within a factor of 2 (Fiorini and Joret 2012).

The BMEP was introduced in the literature on molecular phylogenetics by Desper and Gascuel (2002), based on an estimation model proposed more than 20 years ago by Pauplin (2000). Subsequently, the problem was the subject of extensive research efforts focused on the characterization of its statistical consistency (Desper and Gascuel 2004, Gascuel 2005, Gascuel and Steel 2006) and heuristics (Gascuel 2005, Pardi 2009, Fiorini and Joret 2012, Lefort *et al.* 2015) to tackle and solve its instances.

This article further adds to the above literature by presenting a novel heuristic, called *PhyloES*, that defines the new reference in approximating the optimal solutions to the BMEP. The current state-of-the-art heuristic for the BMEP is FastME 2.0 (Lefort *et al.* 2015), whose algorithmic core is constituted by a local search that deterministically explores (part of) the solution space of the problem by means of topological changes [called *tree rearrangements*Gascuel (2005)] carried out on an initial phylogeny. FastME often proves fast and accurate in practical phylogenetic analyses as well as able to scale to very large molecular datasets. However, its deterministic exploration strategy of the solution space may be too restrictive in some circumstances, causing premature convergence to solutions that can be arbitrarily far from the optimum. PhyloES proposes a possible way around this problem that consists in making nondeterministic the search in the solution space of the BMEP. This task is achieved by combining classical local search strategies with the *Evolution Strategy* (ES) framework discussed in Bäck (1996). Specifically, starting from an initial set of phylogenies, PhyloES first generates a new set of solutions to the problem by using local search strategies similar to those implemented in FastME. Subsequently, PhyloES stochastically recombines the new phylogenies so obtained by means of the so-called *ES operators* (see Section 2.2). The two phases, the iteration of the local search and the recombination, allow spanning the whole solution space to the BMEP by enabling the potential convergence to the optimum on a sufficiently long period. PhyloES can be downloaded at https://github.com/andygaspar/PHYLOES. It is released to the scientific community under the form of a user-friendly open-source Python library, and makes an extended usage of Pytorch (enabling a parallel CUDA GPU implementation of the algorithm discussed in the next sections) to improve computational efficiency.

## 2 Materials and methods

### 2.1 Topological tree rearrangements

The heuristics described in the literature on the BMEP can be classified into two main categories: *constructive* and *agglomerative* (Lemey *et al.* 2009). The constructive heuristics build a phylogeny for a given set *X* of n≥3 taxa by starting from a star-tree that connects a subset of any three taxa in *X* and by iteratively adding the remaining taxa on an edge selected according to a specified criterion. The agglomerative heuristics, instead, akin clustering techniques, construct a phylogeny for *X* by starting from a star-tree that connects all the *n* taxa in *X* and by iteratively aggregating pair of taxa according to a specific selection criterion (Sokal 1958, Saitou and Nei 1987).

FastME leverages powerful local search operators defined by tree rearrangement moves to improve on the solutions found by the above methods. In particular, FastME first generates a feasible solution to an instance of the BMEP by means of an *initial heuristic* and then iteratively improves it by using tree rearrangements, until the length of the best-so-far tree stops improving. FastME allows the use of several *Standard Initialization Algorithms (SIA)* to generate the initial phylogeny. Examples include constructive heuristics that use *Greedy Balanced Minimum Evolution algorithm* (GBME) and *Ordinary Least-Square for Minimum Evolution* (OLSME) as an edge selection criterion (Rzhetsky and Nei 1992, Desper and Gascuel 2002) and agglomerative heuristics based on the *Neighbor-Joining* (NJ) algorithm (Saitou and Nei 1987), the *Unweighted Neighbor-Joining* (UNJ) algorithm (Gascuel 2002), and Gascuel’s BioNJ (Gascuel 1997).

**Figure 1.**
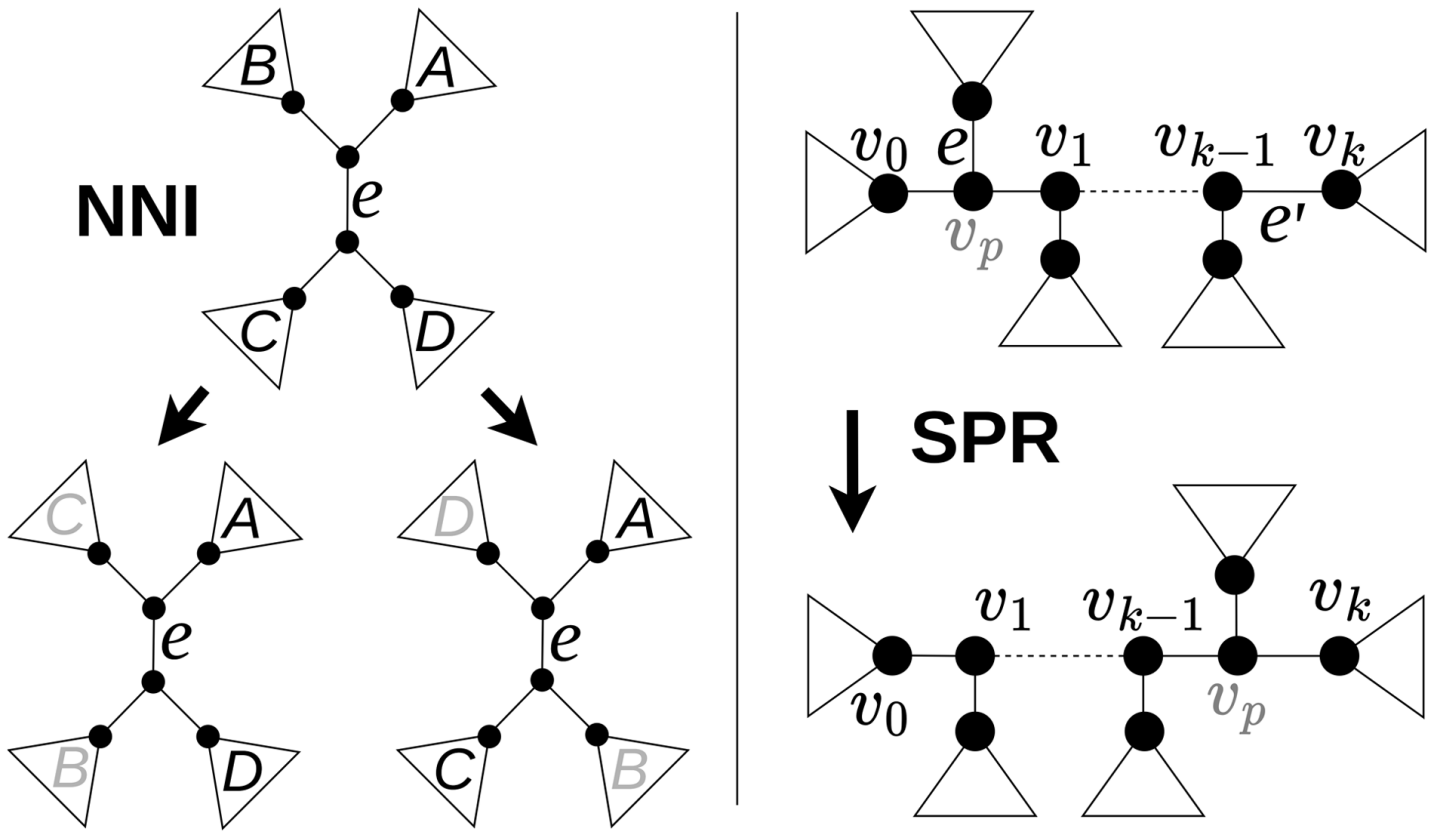
(Left) Representation of an NNI move. When edge *e* is selected, two new trees are created by swapping subtrees A–D and A–C, respectively. (Right) Representation of an SPR move. When edge *e* is selected, the corresponding subtree is pruned from the initial phylogeny and then regrafted onto edge $e^{\prime }$.

In the improvement phase, FastME uses two tree rearrangements, called the *Balanced Nearest Neighbours Interchanges* (BNNI) (Desper and Gascuel 2002) and the *Balanced Subtree Pruning and Regrafting* (BSPR) (Hordijk and Gascuel 2006), which have been shown to provide important consistency properties within the BMEP framework (Bordewich *et al.* 2009). The BNNI and BSPR are based, respectively, on the *Nearest Neighbours Interchanges* (NNI) and *Subtree Pruning and Regrafting* (SPR) tree rearrangements that Lefort *et al.* (2015) use along with formulas that allow to speed up the evaluation of neighbourhood elements thus making FastME extremely computationally efficient. The NNI operation consists of interchanging two subtrees that are adjacent to the same internal branch (Semple *et al.* 2003). The left side of Fig. 1 shows two neighbours that can be obtained by applying NNI to branch *e*: in the first case, the subtree A is swapped with C, while in the second with D. The other possible combinations of exchanges are not considered since they yield *symmetric phylogenies*, i.e. phylogenies topologically equivalent to the ones already taken into account. The SPR neighbours are instead obtained by *pruning* (i.e. removing), a subtree from the initial phylogeny and by *regrafting* (i.e. reattaching), the pruned subtree into one of the remaining branches in the phylogeny (Semple *et al.* 2003). The right side of Fig. 1 shows an example of an SPR move: the subtree defined by edge *e* is first pruned and then reattached into $e^{\prime }$.

Given a phylogeny *T* of *X*, the NNI (SPR) neighbourhood is constituted by all of the trees that can be obtained from the application of a single NNI (SPR) move to each of the internal branches of *T*. As a consequence, the space complexity of the NNI and SPR neighbours is O(n) and $O(n^{2})$, respectively. In the BNNI and BSPR algorithms, the above operators are applied iteratively, by starting from an initial tree *T* and by selecting at each iteration the best neighbour as the initial tree for the next one. Desper and Gascuel (2002) and Hordijk and Gascuel (2006) provide very efficient approaches to evaluate the change in tree length of all moves (O(n) for NNI neighbours and $O(n^{2})$ for SPR neighbours).

### 2.2 Evolution strategies

*Evolution Strategy* (ES), which was first introduced in Rechenberg (1973), is a particular case of *Evolutionary Algorithms* (EA), a class of metaheuristics that are inspired by natural processes and often used to solve complex real-life optimization problems. ESs, as most of the EAs, follow evolutionary rules that draw inspiration from biological phenomena: starting from an initial population they define ways to generate new individuals (*offspring*) through an evolutionary process at each iteration, called a *generation*, and a criterion to define the next generation of individuals (*selection*). These operations are repeated at each generation, by evaluating individuals according to a *fitness function* that represents the quality of a candidate solution, until a stopping criterion is met (Luke 2013). In particular, in a (μ+λ)-ES, the population is initialized by randomly sampling λ individuals; then, by means of a *mutation operator* (i.e. a procedure to convert a single individual into a new one by random changes) λ/μ children are created for each of the parents. Subsequently, the parents and the generated offspring undergo a so-called *truncation selection*, where only the μ fittest individuals survive and form the next generation while the rest are discarded. The process is then repeated with the previous generation’s best individuals as the new parents.

Although EAs have been extensively applied in the literature on ML estimation (Matsuda 1996, Lewis 1998, Brauer *et al.* 2002, Felsenstein 2004, Poladian and Jermiin 2006, Zwickl 2006, Helaers and Milinkovitch 2010), to the best of our knowledge, the algorithm that we present here is the first application of ES for distance-based phylogeny estimation, in particular for the BMEP.

### 2.3 Tree encoding

**Figure 2.**
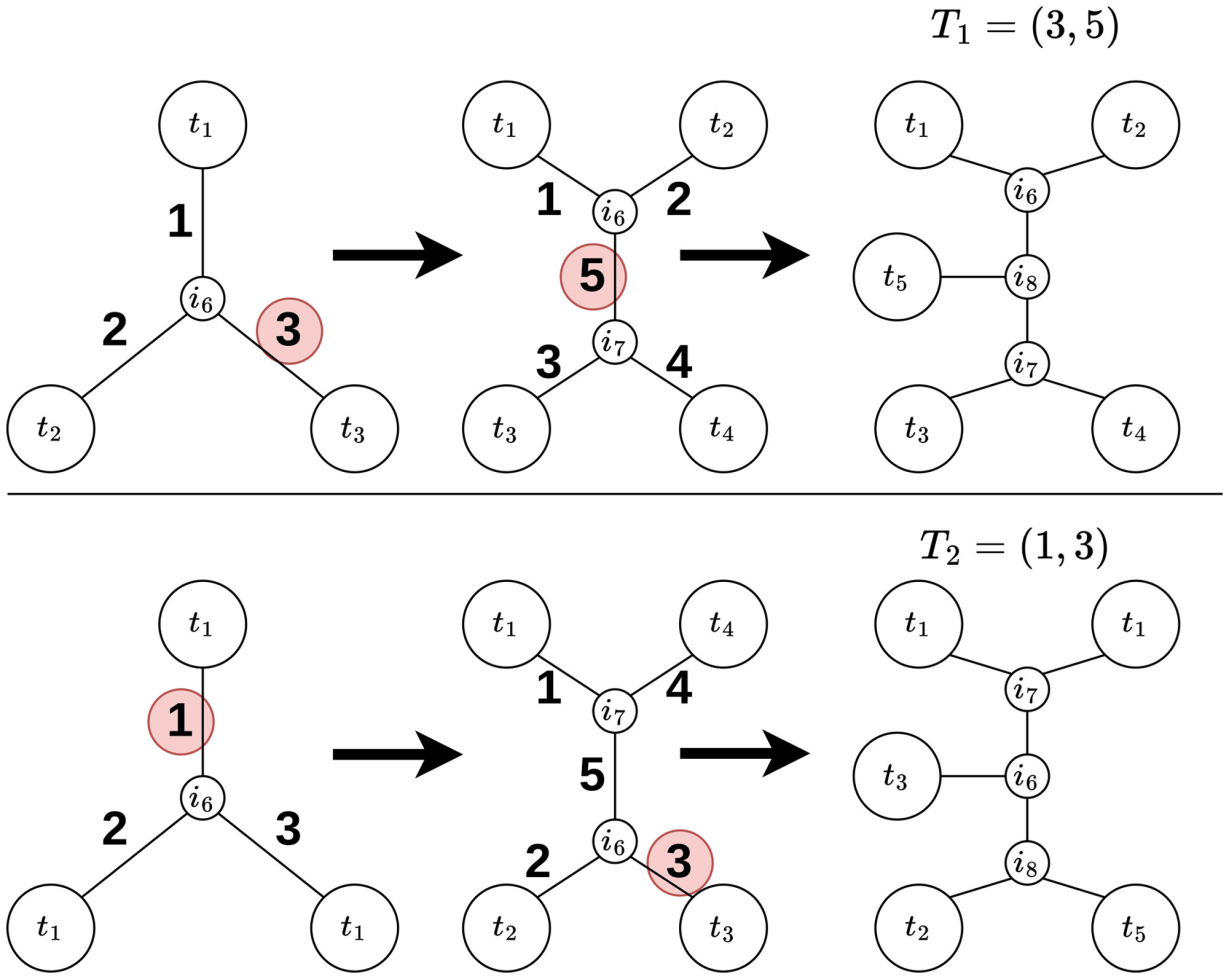
Two examples of tree encoding. (Top) The ordered edge list at the initial step is $E=\{(t_{1},i_{6}),(t_{2},i_{6}),(t_{3},i_{6})\}$. The edge selected for the insertion of $t_{4}$ is $\left(t_{3},i_{6}\right)$, which is the 3-rd in the list. At the next step *E* becomes $\left\{(t_{1},i_{6}),(t_{2},i_{6}),(t_{3},i_{7}),(t_{4},i_{7}),(i_{6},i_{7})\right\}$; $t_{5}$ is then inserted in the edge $t_{5}$ is $\left(i_{6},i_{7}\right)$, 5-th in the list, so the resulting code is $T_{1}=(3,5)$. (Bottom) The initial edge list is $E=\{(t_{1},i_{6}),(t_{2},i_{6}),(t_{3},i_{6})\}$. $t_{4}$ is inserted in $\left(t_{1},i_{6}\right)$, 1-st in the list. At the next step *E* becomes $\left\{(t_{1},i_{7}),(t_{2},i_{6}),(t_{3},i_{6}),(t_{4},i_{7}),(i_{6},i_{7})\right\}$; the edge selected is $\left(t_{3},i_{6}\right)$, 3-rd in the list, providing the resulting code is $T_{2}=(1,3)$.

In order to develop efficiently our ES algorithm we made use of an *ad hoc* representation of the phylogenetic trees inspired by the work of Rohlf (1983). The encoding exploits the fact that any phylogeny can be constructed in a step-wise fashion, starting from an initial star tree composed of only three taxa and a single internal node, and adding one by one all the remaining taxa by selecting at each step an insertion edge (see Fig. 2). If an edge labelling is well defined it is possible to represent a tree with the edge selected for its construction. Formally: we encode any tree of *n* taxa with a vector $\left(h_{1},\ldots ,h_{n-3}\right)$ of n−3 elements, where $h_{i}$ represents the label of the edge selected at the i−th step for the insertion of the (i+3)-th taxon. In our case, for a tree *T* with taxa $t_{1},\ldots t_{n}$ and internal nodes $i_{n+1},\ldots i_{2n-3}$ where $i_{k}$ is the internal node inserted at the *k*-th step of the tree construction, the label of an edge is defined with its index in the edge list *E*, which is unique as we order the elements of *E* first according to the lowest index of the two nodes defining each edge, and in case of ambiguity according to the greatest.

The proof of the consistency of the encoding as well as the details of the coding-decoding algorithms can be found in the Supplementary Material.

The advantage of using such an encoding approach is that it provides a straightforward method for the random generation of phylogenies, and it allows the tree manipulation operating directly on the encodings, by ensuring that when a component of the code of a phylogeny is altered, the result is still a phylogeny. In addition, this encoding turns out to be computationally efficient as the coding and decoding algorithm both require O(n log(n)) operations (proofs in the Supplementary Material).

### 2.4 Phylogenetic inference with ES

To harness the exploration power of ES (Section 2.2) and the efficiency of the BNNI and BSPR tree rearrangement algorithms (Section 2.1) in this section we describe a novel heuristic, PhyloES, specifically designed for the BMEP. PhyloES is an ES, that introduces crucial modifications to the usual (μ+λ)-ES framework. In fact, in our heuristic we foster an efficient exploration of the search space by using tree rearrangement operators to perform *local search* on all the newly generated children, with the aim of steering the mutation process in order to obtain better solutions. In addition, to maintain a good balance between exploration and exploitation, represented respectively by the new and the old individuals, μ and λ are both set equal to the population size. Algorithm 1 illustrates PhyloES and the following paragraphs describe in detail each of its key components.

Algorithm 1: PhyloES

**Required:**μ, ${\text{max}}_{\text{iter}}$, *tol*

 P←{}

 **for**λ times **do**

  $p_{i}\leftarrow \text{random phylogeny}$

  $p_{i}\leftarrow \text{RearrangeTree}(p_{i})$

  $P\leftarrow P\cup \{p_{i}\}$

 **end for**

 Best ← BestIndividual(P)

 Worst ← WorstIndividual(P)

 g←0

 **while**|P|>1 and $g<{\text{max}}_{\text{iter}}$ and L(Worst)−L(Best)>tol**do**

  Q←P

  **for** each $p_{i}\in P$**do**

   $q_{i}\leftarrow \text{GenerateTree}(P)$

   $q_{i}\leftarrow \text{RearrangeTree}(q_{i})$

   $Q\leftarrow Q\cup \{q_{i}\}$

  **end for**

  P← the μ individuals in *Q* whose fitness function are smallest

  P←IndividualReplacement(P)

  Best ← BestIndividual(P)

  Worst ← WorstIndividual(P)

  g←g+1

 **end while**

 **return** Best

*Population initialization*: The population is initialized by sampling μ random phylogenies and applying both BNNI and BSPR to each tree. Furthermore, to foster the initial exploration of the search space we employ a population size decreasing over time according to a halving strategy (see Hallam et al. 2010).

*Offspring*: In the usual ES algorithm blueprint described in Section 2.2, the mutation operator is used to generate new offspring. Mutation yields individuals by introducing random modifications in the encoding of a member of the previous generation’s population. Since PhyloES aims to exploit the similarities between trees resulting from the BNNI and BSPR, the mutation operator is replaced by a *tree generation* operator which takes into account not just a single member but all trees in the population and uses their encoding to construct a new tree. More in detail: each element *j* of a new individual’s encoding is determined by sampling from the set composed by the *j*-th element of all the trees of the previous generation. In mathematical terms, let $T^{g}=\{T_{1}^{g},\;\ldots ,\;T_{k}^{g}\}$ be the set of *k* trees with *n* taxa which represent the population at generation *g*, and let $e(T_{i}^{g})=(h_{i1}^{g},\;\ldots ,\;h_{in-3}^{g})$ be the encoding of $T_{i}^{g}$, with $h_{ij}$ its *j*-th component. Then, a mutated individual $T_{i}^{g+1}$ is obtained from $T^{g}$ by sampling each of its components as


(3) $$h_{ij}^{g+1}=\text{random sample from}\;\{h_{1j}^{g},\;\ldots ,\;h_{kj}^{g}\}.$$

After offspring have been generated, local search is employed to improve the new phylogenies.

*Fitness function*: The fitness is simply defined as the BMEP tree length in Equation (1).

*Selection and stop criteria*: In PhyloES truncation selection is performed as described in Section 2.2: the μ individuals with the lowest fitness value (BMEP length function value) among the previous population and the newly created offspring become the next generation. Regarding the stop criteria, we consider three possibilities: (i) *convergence*, the algorithm runs until all the individuals in the population are identical; (ii) *maximum iterations*, a fixed number of iterations (${\text{max}}_{\text{iter}}$) is performed, which is the most common approach in evolutionary computation; and (iii) *tolerance*, execution stops when the difference, in terms of tree length, between the current best and worst individuals in the population is lower than a given threshold *tol*.

### 2.5 Individuals replacement

In the procedure described above, the simple truncation selection of the best μ individuals within *P* to define the new generation sometimes leads the algorithm to suffer from ‘stagnation’, i.e. the situation in which the new generation remains identical to the previous one, consequently slowing down convergence. Furthermore, this phenomenon tends to occur when the population *P* has multiple instances of its worst individual, which increases the probability of replicating it and decreases the chances of producing better individuals. To overcome this issue, we introduced a simple adjustment on top of the usual truncation selection criterion, similar to the one introduced by Bartoli *et al.* (2019). The authors propose to reduce the number of duplicate individuals in the population by modifying the reproduction phase: whenever they generate a new individual, they check if it is unique in the merged set of parents and already generated offspring; if not, they drop it and generate a new individual. Instead, at each generation of PhyloES, we check in the set *P* for multiple occurrences of the worst individual in terms of tree length and, if any, we replace one of them with a copy of the second worst element. This modification helps avoid stagnation as, if no improvement in the population is gained after one generation cycle, it favours a gradual shift to the replication of the best individual and drives the algorithm towards reaching the convergence stop criteria. The detail of the *IndividualReplacement* algorithm can be found in the Supplementary Material.

### 2.6 Implementation details

The PhyloES Python interface provides not only a simple tool to set up and run phylogenetic analyses but also allows efficient exploitation of computational resources. In particular, the algorithm can be conceptually divided into two main tasks: the ES handling and the local search computations. The former consists of the tree coding and decoding and the offspring operations. All these sub-tasks are characterized by the fact that they can be easily vectorized and performed in batches, i.e. multiple instances can be processed at the same time, allowing the exploitation of GPU resources, particularly suitable for the parallelization of batch vector operations. For this reason, we implemented the entire ES workflow with Pytorch. The latter task instead consists of the BNNI and the BSPR computations: as long as both algorithms make use of tree data structures and perform the tree rearrangement operations via recursive functions, in order to optimize the parallelization we developed a C++ extension of BNNI and BSPR which architectures have been based on the ones provided in FastME, which is called by the python interface via the python-c binding library *ctypes*. The extension allowed us to enable the processing of several trees in parallel that coupled with fast GPU-based encoding and decoding procedures result in a high throughput of BNNI and BSPR operations.

## 3 Results

In this section, we report on the results obtained by running a number of computational experiments on a number of benchmark instances of the BMEP. These experiments were designed so as to answer the following *research questions*: (RQ1) Can PhyloES improve on the solutions found by FastME in terms of tree length? (RQ2) Is the proposed ES approach effective in exploring the search space? (RQ3) How do PhyloES tree solutions differ topologically from those of FastME? (RQ4) Are the observed results reliable from the numerical precision perspective?

In order to evaluate the performances of PhyloES we used as a reference the length of the solutions provided by FastME. Moreover, in order to assess the effectiveness of our evolutionary strategy we compared it versus a pure random initialization approach (RI), consisting of initializing the BNNI and BSPR with random trees.

### 3.1 Experimental setting

In this section, we present the results of our experiments conducted on two benchmark datasets used in Stamatakis (2005) and in Guindon *et al.* (2010), available at https://github.com/stamatak/test-Datasets. More in detail, we extrapolated three datasets, named 100_RDPII (selecting the first 100 taxa from RDPII), 200_RDPII (selecting the first 200 taxa from RDPII) and 300_ZILLA (selecting the first 300 taxa from ZILLA). For each dataset, we generated four distance matrices computed via the FastME software and using respectively the JC69 (Jukes and Cantor 1969), K2P (Kimura 1980), F81 (Hasegawa *et al.* 1981), and F84 (Felsenstein and Churchill 1996) substitution models, always performing pairwise gap removal. The experiments have been run on an Intel Core(TM) i7-10700 (2.90 GHz) 16 cores machine with a GeForce RTX 2070 SUPER GPU. The parameters ${\text{max}}_{\text{iter}}$ and *tol* have been respectively set to 1000 and $10^{-12}$. To favour a wider initial exploration of the search space the population size has been set to 64 for the first 5 iterations, 32 from the 5-th to the 25-th iteration and 16 from the 25-th iteration onward.

In order to ensure a fair comparison, for each instance we tested all the SIA of FastME and the best solution obtained out of the five initialization algorithms was the one taken into account for our analysis. We remark that this procedure was performed only once per instance as regardless of the chosen SIA, FastME is a deterministic algorithm and it does not benefit from multiple runs. Instead, the RI algorithm and PhyloES have been run 10 times for each of the 12 problems and the collected data have been used to produce the analysis and the statistics shown in this section. Furthermore, when comparing PhyloES to the RI, we first run our algorithm and count the number of generated trees, and then we perform the same number of RI iterations.

### 3.2 RQ1, Tree length analysis

In Table 1, we report, for each of the considered datasets, the performance of FastME, PhyloES, and the RI. For FastME, we indicate the BMEP length function value of the best solution found and the SIA that led to the solution. Instead, for PhyloES and the RI, we outline the average BMEP length value of the best solution, its standard deviation, the number of unique and distinct best phylogenies across the different runs and the average improvement with respect to FastME (where a negative value indicates a lower BMEP length value than FastME). The results in Table 1 show that PhyloES, on average, either matches or outperforms FastME in terms of tree length. In the 100 taxa problems, PhyloES achieves a better solution with the F81 and JC69 datasets while in the other cases performs on par with FastME. As the number of taxa increases, the difference between the two methods becomes more significant with an average improvement of 0.04% over FastME. Again, with the F81 and JC69 datasets, there is a larger gap, yet the same can be stated for the RI which probably indicates that these instances are easier to solve by exploring the solution space utilizing the considered local strategies. However, if we consider the single substitution models, there is a clear trend in the improvement which confirms the effectiveness of exploration strategies for large search spaces. This observation is confirmed also by the RI behaviour which, in terms of tree length improvement with respect to the FastME solutions, follows a similar trend. Finally, we report that in all cases in which PhyloES provides multiple solutions over the 10 runs (which number is shown in column *n sol* of Table 1), they are all better than the respective FastME ones, which explains the very low value of their standard deviation (column *Lstd* of Table 1).

**Table 1.** BMEP length function value and number of solutions analysis for FastME, PhyloES, and the RI.

	FastME		PhyloES				RI			
Dataset	Best SIA	*L*	*L* avg	*L* std	*n* sol	improvement %	*L* avg	*L* std	*n* sol	Improvement %
100_rdpii F81	OLSME	**8.415707**	**8.415707**	0.000000	1	0.0000	**8.415707**	0.000000	4	−0.0000
100_rdpii F84	OLSME	8.461857	**8.461710**	0.000000	1	−0.0017	**8.461710**	0.000000	4	−0.0017
100_rdpii JC69	OLSME	**8.406178**	**8.406178**	0.000000	1	0.0000	**8.406178**	0.000000	3	−0.0000
100_rdpii K2P	OLSME	8.453968	**8.453811**	0.000000	1	−0.0018	**8.453811**	0.000000	2	−0.0018
200_rdpii F81	BioNJ	13.987183	**13.985146**	0.000095	2	−0.0146	13.985354	0.000190	10	−0.0131
200_rdpii F84	BioNJ	14.074191	**14.068298**	0.000048	2	−0.0419	14.068800	0.000301	10	−0.0383
200_rdpii JC69	BioNJ	13.973130	**13.971048**	0.000059	2	−0.0149	13.971330	0.000130	10	−0.0129
200_rdpii K2P	BioNJ	14.062630	**14.056749**	0.000031	2	−0.0418	14.057467	0.000368	10	−0.0367
300_zilla F81	NJ	4.482169	**4.480371**	0.000008	3	−0.0401	4.480839	0.000187	10	−0.0297
300_zilla F84	BioNJ	4.506561	**4.504317**	0.000001	2	−0.0498	4.504646	0.000213	10	−0.0425
300_zilla JC69	NJ	4.479926	**4.478132**	0.000000	1	−0.0400	4.478463	0.000200	10	−0.0327
300_zilla K2P	BioNJ	4.501083	**4.498846**	0.000000	1	−0.0497	4.499206	0.000196	10	−0.0417

The boldface represents the minimum length out of the three algorithms.

### 3.3 RQ2, Search space exploration

Comparing PhyloES with the RI we can see that, except for the 100 taxa datasets in which they attain the same performance, the evolutionary approach we propose leads to a clear improvement over the simple RI strategy. Additionally, our method shows more consistency in the final length value with respect to the RI. In fact, from Table 1, it is noticeable that on half the problems PhyloES produces the same final solution throughout the 10 runs whereas, on the other problems, a limited number of different solutions is found. On the contrary, the RI yields more distinct phylogenies in the 100 taxa problems while the 200 and 300 taxa datasets show no consistency at all, providing at each run a different solution. Furthermore, we can also inspect this phenomenon by turning our attention to the standard deviation of the best length value attained by each method. In those cases where PhyloES leads to multiple solutions, their tree length lies in a very small interval, hence a standard deviation in the order of $10^{-5}$. Conversely, the RI standard deviation tends to be an order of magnitude greater, which indicates a larger variability in the quality of the results.

To conclude, we also report that, except for a single run on the 200_RDPII F81 dataset in which the algorithm stopped due to the minimum tolerance criteria, in all the remaining runs PhyloES stopped due to convergence.

**Figure 3.**
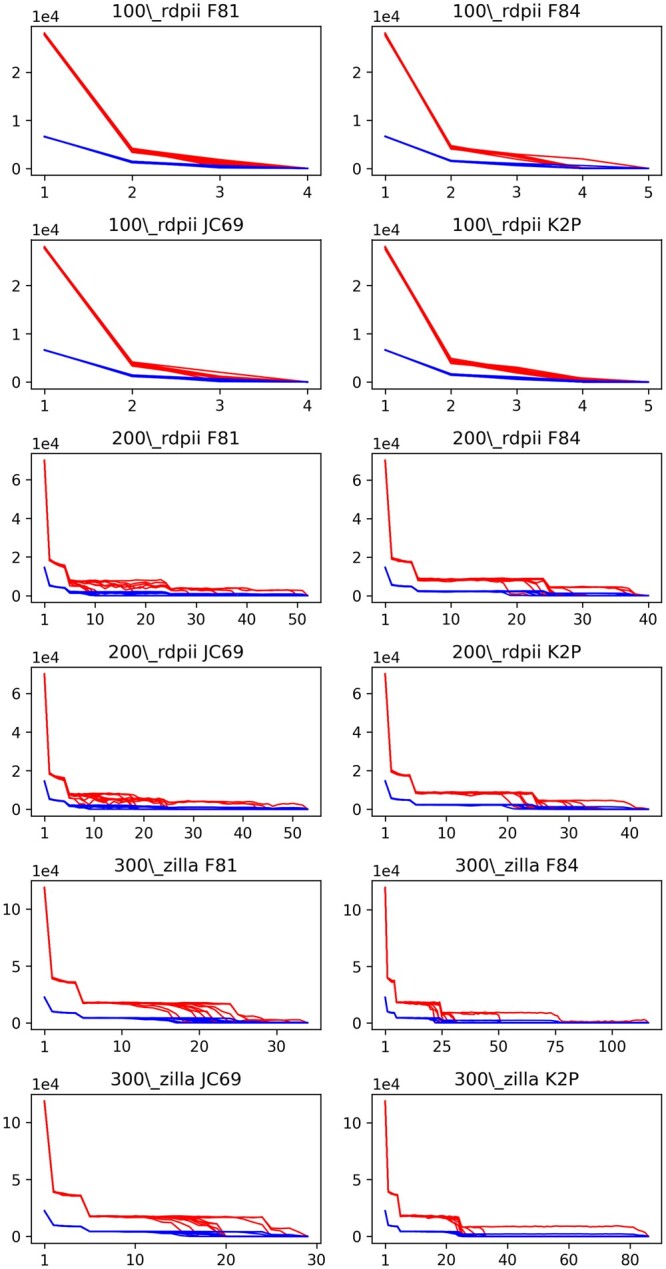
Number of BNNI (red line) and BSPR (blue line) iterations per generation in PhyloES.

To analyse the scalability of our approach, we study the dependency of the computational effort required by PhyloES, with respect to the instance size, and its variability, as well as how it compares with the RI. It is worth mentioning that since PhyloES consists of multiple runs of BNNI and BSPR it cannot outperform FastME in terms of computational time. In Table 2, we detail, for each problem instance, the number of trees evaluated by both algorithms and for each one we also indicate the average and standard deviation of the execution time (in seconds), the average total number of NNI and SPR calls per run as well as the average number of generations carried out by PhyloES. The FastME computation time is reported in the last column of Table 2. Instead, in Fig. 3, we display the trend of the PhyloES’s number of NNI and SPR calls along the generations. From Table 2, we see how PhyloES significantly outperforms RI also in terms of computational time, showing a lower average time to solution and a smaller standard deviation. A straightforward explanation for such a difference can be found in the number of NNI and SPR calls reported in the table. On average, given the same amount of initial trees, PhyloES performs fewer iterations of the local search operators with respect to RI resulting in a much smaller computation time even though there is overhead due to the ES approach, *id est* the coding, decoding and tree recombination phase. The reason for this phenomenon lies in the fact that at each generation, the initial population is composed of individuals that are the recombination of previous BNNI and BSPR neighbours hence they are much more likely to be closer to optimum (lower tree length) compared to a set of randomly generated trees, as in the case of the RI initial trees. This fact is even more evident from the curves in Fig. 3, in which we can notice how, for all datasets, the number of BNNI and BSPR iterations drops immediately after the first generation and then gradually decreases at each generation. These results show the effectiveness of the ES we adopt, by employing an evolutionary approach PhyloES is able to efficiently explore the solution space by requiring fewer NNI and SPR iterations.

**Table 2.** Comparison of the computational time in seconds and the number of NNI and SPR calls in PhyloES and the RI.

		PhyloES					RI				FastME
Dataset	*n* trees	Avg time	Time std	Iterations	nni	spr	Avg time	Time std	nni	spr	Time
100_rdpii F81	256, 0	1, 7	0, 0	4, 0	32.852, 5	8.364, 1	2, 8	0, 1	111.432, 2	26.462, 1	0, 2
100_rdpii F84	275, 2	2, 0	0, 2	4, 3	34.978, 8	9.073, 4	3, 6	0, 4	119.981, 3	28.477, 1	0, 2
100_rdpii JC69	256, 0	1, 7	0, 1	4, 0	32.613, 4	8.286, 4	3, 0	0, 3	111.477, 9	26.468, 5	0, 2
100_rdpii K2P	281, 6	2, 0	0, 2	4, 4	34.992, 1	9.044, 9	3, 7	0, 4	122.765, 2	29.141, 5	0, 2
200_rdpii F81	660, 8	48, 6	18, 5	27, 3	241.433, 8	60.035, 5	83, 5	28, 9	589.174, 6	122.665, 9	1, 4
200_rdpii F84	632, 0	63, 8	7, 5	31, 7	320.680, 4	81.820, 5	85, 2	18, 3	592.632, 8	123.768, 6	1, 4
200_rdpii JC69	683, 2	51, 9	13, 4	28, 9	254.344, 9	63.662, 8	95, 3	27, 9	664.862, 5	138.599, 6	1, 3
200_rdpii K2P	588, 8	64, 0	6, 2	31, 8	321.755, 8	82.422, 4	79, 9	14, 3	553.963, 2	115.777, 8	1, 4
300_zilla F81	636, 8	224, 4	24, 3	27, 0	550.774, 2	125.557, 9	377, 9	87, 4	1.059.653, 6	200.291, 1	4, 4
300_zilla F84	748, 8	280, 3	80, 5	39, 0	690.211, 0	157.725, 7	484, 7	279, 9	1.370.971, 3	259.037, 1	4, 4
300_zilla JC69	718, 4	210, 7	30, 0	22, 4	524.163, 3	119.632, 6	383, 1	68, 8	1.077.795, 2	203.781, 7	4, 5
300_zilla K2P	620, 8	271, 2	72, 4	33, 8	671.155, 2	154.428, 8	353, 9	197, 8	1.000.720, 6	189.174, 5	4, 3

### 3.4 RQ3, Topological structure analysis

To further investigate the difference of the solutions provided by PhyloES and FastME we now focus our attention on the quality of the obtained solution trees. More in detail we aim to analyse if and how much the solutions differ by use of the Robinson–Fould (RF) Distance (Robinson and Foulds 1981), which defines the distance between two phylogenetic trees as the minimum number of edit operations, edge contractions or/and edge extensions, needed to convert one into the other. In Table 3, we indicate the RF distance between the best solution found by FastME and PhyloES as well as the number of different solutions found by PhyloES. The reported values clearly show how the RF distance tends to increase with the number of taxa. In particular, for the 200 and 300 taxa cases, its high-value range (64–74) indicates a significant difference in terms of tree topology. This analysis confirms the added value of the evolutionary approach we developed suggesting that the observed relatively small changes in tree length (in this case around the order of $10^{-4}$) imply notable differences in terms of the resulting tree structure. Hence the impact of the strategy employed in PhyloES leads to a significant difference in terms of the phylogenetic inference problem we aim to solve.

**Table 3.** Topological RF distances between FastME and PhyloES solutions.

Dataset	Avg RF	Std RF	*n* solutions
100_rdpii F81	0.00	0.00	1
100_rdpii F84	6.00	0.00	1
100_rdpii JC69	0.00	0.00	1
100_rdpii K2P	6.00	0.00	1
200_rdpii F81	44.40	2.06	2
200_rdpii F84	67.80	2.89	2
200_rdpii JC69	45.60	1.26	2
200_rdpii K2P	66.60	1.89	2
300_zilla F81	82.80	12.83	3
300_zilla F84	62.80	5.43	2
300_zilla JC69	74.80	1.03	1
300_zilla K2P	60.80	1.03	1

### 3.5 RQ4, Numerical precision analysis

In principle, the BMEP might suffer from numerical precision issues, a problem which should be carefully taken into account. In fact, with a number of taxa approximately greater than 50, some of the terms of Equation (1) might take values below the machine epsilon precision. This obstacle, on one hand clearly represents a theoretical limitation of the BMEP, as it does not allow to guarantee a correct comparison between trees in terms of tree length, on the other, it turns out to have quite a small impact in real applications. In fact, if we consider a phylogeny *T* of *n* taxa, n≥50, and with a given topology τ, we can split its length [Equation (1)] in two terms:


(4) $$\begin{array}{c} L(T)=\sum_{i,j\in X}2^{1-\tau_{ij}}d_{ij}=\sum_{i,j\in A}2^{1-\tau_{ij}}d_{ij}+\sum_{i,j\in B}2^{1-\tau_{ij}}d_{ij}\\ =l_{A}(T)+l_{B}(T) \end{array},$$

where $A:=\{\tau_{ij}\in \tau |\tau_{ij}<50\}$ and $B:=\{\tau_{ij}\in \tau |\tau_{ij}\geq 50\}$. For the right-hand side $L_{B}(T)$ of Equation (4), which represents the summation of the terms affected by numerical precision issues, we can easily derive the following inequality:


(5) $$\begin{array}{c} l_{B}(T)=\sum_{i,j\in B}2^{1-\tau_{ij}}d_{ij}\leq \sum_{i,j\in B}2^{1-\tau_{ij}}\underset{i,j\in X}{\max}d_{ij}\\ \leq |B{|}^{2}2^{1-50}\underset{i,j\in X}{\max}d_{ij}<n^{2}2^{-49}\underset{i,j\in X}{\max}d_{ij}=\omega , \end{array}$$

in which if the elements $d_{ij}$ are not all identical, as in most of the real applications, the first inequality can be turned into a strong inequality. The last strong inequality instead, holds due to the fact that |B|<n, as an unrooted binary tree must have at least two cherries, where cherry indicates a pair of leaves with a mutual topological distance equal to 2. The value ω of Equation (5) represents a bound for the contribution of the terms of *B* in L(T) and it can be used as an error tolerance for the trees’ length comparison. In other words, once defined $\tilde{L}$ as the numerical computation of *L* and given two phylogenies $T_{1}$ and $T_{2}$, Equation (5) allows to state that:


(6) $$\tilde{L}(T_{1})+\omega <\tilde{L}(T_{2})\Rightarrow L(T_{1})<L(T_{2}).$$

In Table 4, we show the ω values computed for the different datasets used in the experimental phase. As long as the improvements provided by PhyloES with respect to the FastME solution (Table 1) are within $10^{-5}$ and $10^{-6}$ we can certify that inequality (6) is satisfied and therefore safely confirm the robustness of our results. Furthermore, in our experiments all the solution trees provided by each of the tested algorithms were characterized by a maximum topological distance between taxa lower than 50.

**Table 4.** Values of the error tolerance for the considered datasets.

Taxa	Dataset	F81	F84	JC69	K2P
100	rdpii	9.64E−12	9.77E−12	9.60E−12	9.74E−12
200	rdpii	4.03E−11	4.07E−11	4.01E−11	4.06E−11
300	zilla	2.52E−11	2.56E−11	2.52E−11	2.55E−11

## 4 Conclusions and future works

We introduced here a novel ES approach for the BMEP able to outperform FastME in practical phylogenetic analyses. Computational experiments showed that, at the expense of some additional computational time, the use of local search operators like BNNI and BSPR running multiple times under a well-designed exploration framework leads consistently to solutions that improve on the ones given by FastME.

We have also analysed how small reductions in the tree length may result in quite different topologies, hence in relevant variations from the phylogenetic inference perspective. By studying the behaviour of the proposed algorithm across several runs, we observed that it is stable, despite its stochastic nature. In fact, for all the considered problem instances, a single or limited set of unique solutions was found over multiple runs on the same instance. Furthermore, by analysing the numerical precision issues of the BMEP, we provided an inequality which gives a numerical tolerance for the exact comparison of two trees’ length, allowing us to certify the robustness of the solutions provided by PhyloES.

In its present implementation, the scalability of PhyloES is strongly limited by the computational effort required by the BNNI and BSPR algorithms, limiting its application on larger instances without requiring high-performance computing resources. However, the large amount of biological data available and the recent huge progress of Machine Learning techniques (Azouri *et al.* 2021), suggests that the two local search algorithms might be replaced with some appropriate learned approximations, capable of evening out or improving their performance in terms of solutions while drastically reducing the computational effort, potentially enabling PhyloES to be employed on larger instances. Finally, it would be interesting to further investigate the possibility of applying the PhyloES framework to other phylogenetic criteria, such as Maximum Likelihood or Maximum Parsimony. PhyloES partially owes its efficiency to the BNNI and BSPR which are particularly fast due to the exploitation of the mathematical properties of the BMEP. A natural question, therefore, is whether the combinatorics and the optimization aspects of other criteria may be exploited to reach a similar efficiency. Addressing this interesting question definitely warrants additional research effort.

## Supplementary Material

btad660_Supplementary_Data

## Data Availability

The code and data underlying this article are available at https://github.com/andygaspar/PHYLOES.
