# Supplementary material for: An evolution strategy approach for the balanced minimum evolution problem
Source: Bioinformatics. 2023 Oct 27;39(11):btad660. doi: 10.1093/bioinformatics/btad660 (PMC10641104; doi:10.1093/bioinformatics/btad660)
Supplement: btad660_Supplementary_Data [file btad660_supplementary_data.pdf]

Supplementary material for An evolution strategy approach for the Balanced Minimum Evolution Problem

Andrea Gasparin, Federico Julian Camerota Verdù,
Daniele Catanzaro and Lorenzo Castelli

1 PhyloES encoding detail

PhyloES requires the definition of an appropriate encoding to represent the individuals. The problem of encoding trees has been widely studied in the literature (Caminiti *et al.*, 2007; Catanzaro and Pesenti, 2019) and several tree codes have been proposed (Prüfer, 1918; Neville, 1953). However, the encoding method proposed in Rohlf (1983) is specific for unrooted binary trees and it offers two main advantages: it provides a straightforward method for the random generation of phylogenies, and it allows to manipulate trees operating directly on the encodings, by ensuring that when a component of the code of a phylogeny is altered, the result is still a phylogeny. Rohlf (1983)'s encoding defines a map ϕ between the edge set E of a tree and the set of coding vectors H whose elements are of the form $(h_1, \ldots, h_{n-3})$, where n is the number of leaves and $h_k \in \{1, 2, \ldots, 2k-3\}$. Such a function ϕ is proven to be well defined and bijective as long as a well defined labelling of the edges is given (Rohlf, 1983).

1.1 Internal edge labels

For our encoding, we first assume to work with an ordered set X of n taxa, $t_1, t_2, \ldots, t_n$. We define the label of an edge as its index in the edge list E of a phylogenetic tree, which is ordered first according to the lowest index of the two nodes defining each edge, and in case of ambiguity according to the greatest. To finally guarantee the uniqueness of the edge labels we have to ensure that a well defined labelling of the internal nodes is given. We achieve so by considering the step-wise tree construction method that, starting from an initial star tree composed of the first three taxa and a single internal node, adds one by one all the remaining taxa. All internal nodes are labelled according to their insertion order, starting from the index $k = n + 1$ up to the index $k = 2n - 2$ (where n is the number of taxa).

1.2 Decoding

ϕ^{-1}, the decoding map described in algorithm 1, allows us to retrieve the edge set of a tree T of n taxa given its code h. Since in our case h represents the instructions for the construction of T we simply need to build E step-by-step according to h, starting from the initial star composed by the first three taxa. At each step k we remove the selected edge h_k, we add the two new edges and we fix the order of E. Algorithm 1 shows the details of the decoding procedure, which we will now analyse in depth. From now onwards we will indicate a taxa with the letter t and internal nodes with the letter i.

At the initial step $E = \{(t_1, i_{n+1}), (t_2, i_{n+1}), (t_3, i_{n+1})\}$; h, which elements are in $\{1, 2, 3\}$, indicates the index in E of the edge selected for the insertion of t_4 and the internal node i_{n+2}. Referring to the example of Figure 1, in which $h = (2, 5, 1)$, the edge selected is $E[2] = (t_2, i_7)$. The function **InsertTaxonEdge** (algorithm 2) adds the new edge in the list after the only edge connecting the previous taxa t_3, (t_3, i_7), obtaining $E = \{(t_1, i_7), (t_2, i_7), (t_3, i_7), (t_4, i_8)\}$. This insertion preserves the correct ordering of E since the edges having taxa as first component, $(t_*, *)$, always come before the others, which are of the form (i_α, i_β). This is always true as we established that taxa are indexed from 1 to n and internal nodes form $n + 1$ to $2n - 2$.

Then, the selected edge (t_3, i_7), has to be removed and its nodes will be connected with the new internal node i_8 forming two new edges. Therefore, (t_3, i_7) can be simply replaced by (t_3, i_8), (line 5 in algorithm 1). In this case E is still ordered so no fixing is needed. However, in general it

Algorithm 1 ϕ^{-1}, Decoding

Require: h, $\{t_1, \dots, t_n\}$,
$E := \{(t_1, i_{n+1}), (t_2, i_{n+1}), (t_3, i_{n+1})\}$ (the initial star tree)
for $k = 1, k \leq n - 3, k{+}{+}$ **do**
  $(v_\alpha, i_\beta) = E[h_k]$
  InsertTaxonEdge$((t_{k+3}, i_{n+1+k}))$
  Set $(v_\alpha, i_\beta) = (v_\alpha, i_{n+1+k})$
  FixPosition$((v_\alpha, i_{n+1+k}))$
  Insert$((i_\beta, i_{n+1+k}))$
end for
return E

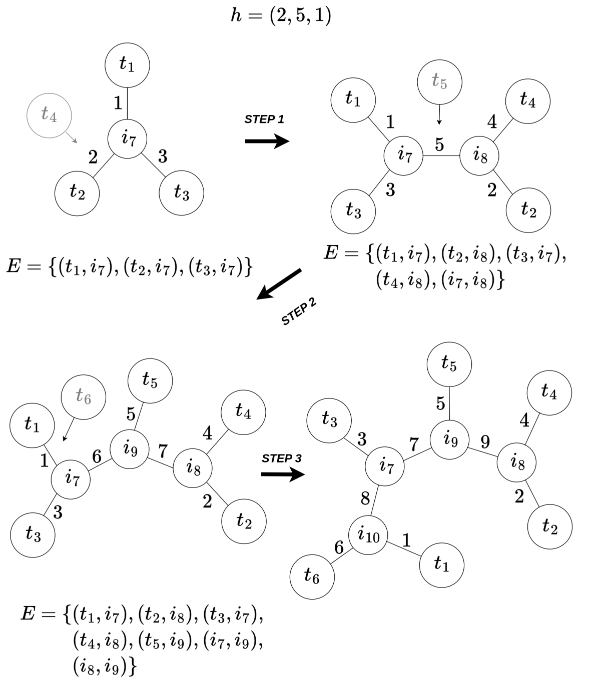

Figure 1: Example of the tree construction and encoding for a phylogeny with six taxa. Starting from a star-tree with three taxa connect to a single inner node, the remaining taxa are added to the tree iteratively and both the edge set E and the encoding H are updated at each addition.

might be necessary to adjust the position of the new edge, task which is handled by the function **FixPosition** (algorithm 3). In fact, as shown in figure 2, if the selected edge (i_α, i_β) is connecting two internal nodes i_α and i_β, and if i_α is linked with some other internal node i_γ with $\gamma > \beta$ and the node $*$ is either a leaf or an internal node with index greater than γ, we have that the order of $E = \{\dots, (i_\alpha, i_{n+1+k}), (i_\alpha, i_\gamma,), \dots\}$ after the replacement, must be fixed swapping (i_α, i_{n+1+k}) and (i_α, i_γ), since $i + 1 + k > \gamma$. In the case in which $*$ is also an internal node with index greater than γ, an additional swap between (i_α, i_{n+1+k}) and $(i_\alpha, *)$ has to be performed.

The last line of the loop of ϕ^{-1} is the insertion in E of the edge (i_β, i_{n+1+k}), handled by the function **Insert** (algorithm 4). Here there are three cases: if $k = 1$, the edge (i_{n+1}, i_{n+2}) is simply added to the end of the list. If $k > 1$ and no edge in E has i_β as first component, (i_β, i_{n+1+k}) must be inserted in E after $(i_\gamma, *)$, where i_γ is the internal node with the greatest index such that $\gamma < \beta$ and $*$ is the

Algorithm 2 InsertTaxonEdge$((t_{k+3}, i_{n+1+k}))$

$(t_{k+2}, *) =$ unique edge connecting leaf t_{k+2}
Insert (t_{k+3}, i_{n+1+k}) in E after $(t_{k+2}, *)$

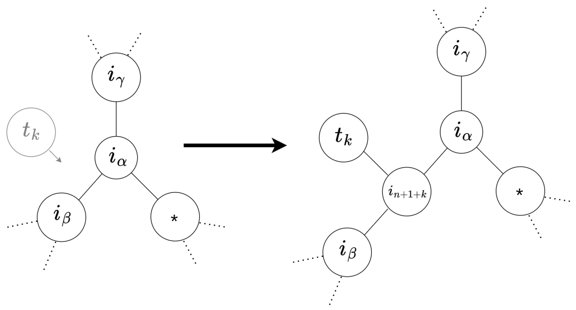

Figure 2: In case of $\gamma < \beta$, the order of E must be adjusted.

Algorithm 3 FixPosition$((v_\alpha, i_{n+1+k}))$

$(v_\alpha, *)$ = last occurrence in E of an edge with first component i_β
insert (v_β, i_{n+1+k}) in E after $(v_\alpha, *)$

---

internal node connected to i_γ with the greatest index. This is the case of (i_8, i_9) in the second step of the example of figure 1; before its insertion, $E = \{(t_1, i_7), (t_2, i_8), (t_3, i_7), (t_4, i_8), (t_5, i_9), (i_7, i_9)\}$; since no edge E has i_8 as first component and i_7 is the internal node with the greatest index lower than 8, (i_8, i_9) is inserted after (i_7, i_9). The last case is the one in which there are in E some edges with i_β as first component. If so, (i_β, i_{n+1+k}) has to be inserted after the last of them, a task performed by function 3.

Algorithm 4 Insert$((i_{n+1+k}, i_\beta))$

1: **if** k=1 **then**
2:     insert (i_{n+1}, i_{n+2}) at the end of E
3: **else if** any $(i_\beta, *)$ not in E **then**
4:     $(i_\gamma, *)$ = edge in E s.t. γ is the greatest index with $\gamma < \beta$
5:     insert (i_β, i_{n+1+k}) after $(i_\gamma, *)$
6: **else**
7:     **FixPosition**$((i_\beta, i_{n+1+k}))$
8: **end if**

1.2.1 Decoding complexity

The decoding algorithm, ϕ^{-1} is composed of a *for* loop of $n-3$ $(O(n))$ iterations. The function **InsertTaxonEdge** (algorithm 2) requires constant time since the position of each node in the list can be traced out and reached in O(1). **FixPosition** (algorithm 3) needs to iterate through E starting from (v_α, i_{n+k+1}) to find the last occurrence of an edge with first component equal to v_α; since any node is linked to at most three other nodes, the function is $O(1)$. Concerning function **Insert** (algorithm 4), apart the one in line 4, all operations require $O(1)$. Since in line 4 the index γ is unknown, it must be searched in an ordered list, an operation that requires $O(\log(n))$ operations. Due to the above reasoning the whole complexity of ϕ^{-1} is $O(n \log(n))$.

1.3 Coding

The coding algorithm, ϕ, maps the edge set of a phylogenetic tree into its coding vector h. We remark that in order to obtain the correct code of a tree two conditions must be ensured:

1. The internal node labels must be consistent with the one chosen for our encoding.
2. E must be ordered primarily according to the edges' first component index and, when equal, according to the second component index.

Concerning 1), we notice that our internal node labels are determined by the phylogeny step-wise tree construction, that is the one followed in the decoding algorithm and for which the label of the internal node inserted at the construction step k is $n+k+1$. Consequently, the edge list E of a phylogeny should always contain the edge (t_n, i_{2n-2}), which is the one added to the list in the last step. If we have instead that t_n is linked to i_α with $i_\alpha \neq i_{2n-2}$ we can fix the inconsistency by swapping the labels of the two internal nodes. If we then remove the edge (t_n, i_{2n-2}) from E we should have that t_{n-1} is linked i_{2n-3}: if this is not the case, we can again fix the inconsistency by performing the index swap between i_{2n-3} and the internal node connected to t_{n-1}. To complete the adjustment of the internal node labels, as described in algorithm 5 this procedure has to be performed for all the remaining steps. We remark that at each step, when we remove the edge (t_k, i_{n+k+1}), the tree must be reconnected. This is done connecting the two nodes v_α and i_β, with $\alpha < \beta$, previously linked to i_{n+k+1} (figure 3).

Algorithm 5 FixInternalNodesLabels

Require: E

for $k = n, k \geq 4, k--$ **do**
    i_γ = internal node connected to t_k
    v_α, i_β = nodes connected to i_γ different to t_k
    if $\gamma = n+k-2$ **then**
        swap indexes between i_γ and i_{n+k-2}
    end if
    Remove (t_k, i_{n+k-2}) and (i_β, i_{n+k-2}) from E
    Set $(v_\alpha, i_{n+k-2}) = (v_\alpha, i_\beta)$
end for
return E

Once ensured condition 1), condition 2) can be guaranteed by sorting E.

The idea behind the coding procedure (algorithm 6) is to invert the flow of the decoding algorithm in a similar fashion to algorithm 5, and to retrieve h deconstructing the tree by removing one-by-one all taxa from t_n to t_4. The main difference with algorithm 5 is that each time that an edge is removed the order of E must be restored. This might happen when reconnecting nodes v_α and i_β, in the case in which v_α is an internal node connected with some other internal node i_γ such that $\gamma > \beta$. In that case, the function 7 ensures the correct ordering by applying the appropriate swaps. Since finally E is correctly ordered, the component h_k is given by the index of (v_α, i_β) in E which is the edge between the nodes v_α and i_β previously connected to i_{n+k+1}.

Algorithm 6 ϕ

Require: E

1: **for** $k = n, k \geq 4, k--$ **do**
2:     v_α, i_β = nodes connected to i_{n+k-2}
3:     Remove (t_k, i_{n+k-2}) and (i_β, i_{n+k-2}) from E
4:     Set $(v_\alpha, i_{n+k-2}) = (v_\alpha, i_\beta)$
5:     **FixPosition2**((v_α, i_β))
6:     h_k = index of (v_α, i_β) in E
7: **end for**
8: **return** E

1.3.1 Coding complexity

The whole coding procedure is composed of three parts: the function **FixInternalNodesLabels** (algorithm 5), the sorting of E and the function *phi* (algorithm 6). **FixInternalNodesLabels** consists of a for loop composed of only O(1) operations, so it requires $O(n)$ operations. Sorting E instead requires $O(n\log(n))$. In the for loop of ϕ all operations but the one of line 6 require $O(1)$; the index retrieval of line 6 requires instead $O(\log(n))$ operations since it is a search in an ordered list. The whole cost of ϕ is therefore $O(n\log(n))$. In conclusion, all three steps of the coding require in total $O(n\log(n))$.

Algorithm 7 FixPosition2((v_α, i_β))

$v_{\gamma1}, v_{\gamma2}$ = two nodes connected to v_α different from i_β, with $\gamma1 < \gamma2$
if $\gamma2 > \beta$ **then**
  swap $(v_\alpha, v_{\gamma2})$ and (v_α, i_β) in E
  if $\gamma1 > \beta$ **then**
    swap $(v_\alpha, v_{\gamma1})$ and (v_α, i_β) in E
  end if
end if

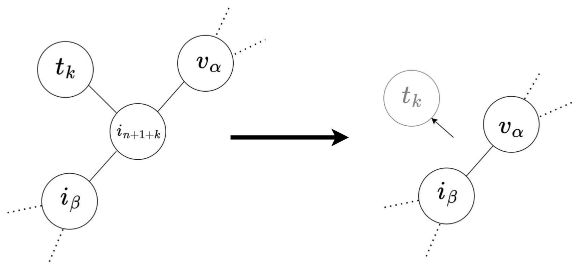

Figure 3: Removal of taxon t_k

1.4 PhyloES encoding advantages

As mentioned here in section 1, the encoding we developed turns out particularly handy to generate and manipulate trees. The decoding algorithm (6) provides in fact a constructive procedure to generate a random tree of n taxa, by sampling a vector of $n-3$ components such that:

$$\begin{aligned} h_1 &= \{1, 2, 3\} \\ h_2 &= \{1, 2, 3, 4, 5\} \\ &\dots \\ h_k &= \{1, \dots, 2k-3\} \\ &\dots \\ h_{n-3} &= \{1, \dots, 2n-6\} \end{aligned} \tag{1}$$

In addition, in the PhyloES offspring phase, a new individual is generated defining its coding vector h, which components are obtained by sampling from the components of the whole population. Since any vector of the form described in equation 1 is a valid tree we are ensured that such a new individual is a valid tree. Figure 4 shows an example of the generation of individuals in the case of a population composed of three trees of eight taxa.

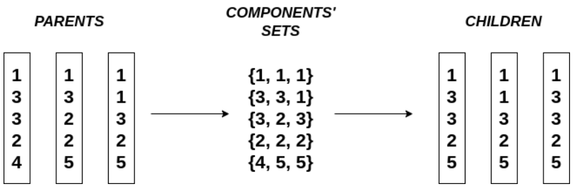

Figure 4: Example of generation of a new individual by sampling each component from the population.

2 Individual replacement

Here we provide the pseudo-code of the individual replacement algorithm used in PhyloES.

To better understand the flow of algorithm 8, let us suppose to have the following population of trees $P = a, b, c, d, e, f, f, f$, where elements are ordered by tree length. The worst individual f has two occurrences, so its first occurrence in P is replaced e, which is the second worst individual, obtaining

Algorithm 8 IndividualReplacement

Require: P, the current tree population
  sort P by BMEP length value
  w = worst individual in P
  if w has multiple occurrences in P **then**
    rep = second worst individual in P s.t. $w \neq rep$
    replace the first occurrence of w with rep
  end if
  return P

$p = a, b, c, d, e, e, f$. In this manner, any new individual is less likely to be similar to f decreasing the chances of generating full or partial replicas of f, a fact which would slow down the convergence of the algorithm. The benefit of this adjustment has been empirically proven in our experiments, but a detailed analysis of the advantages of such a technique is beyond the scope of this article.

References

Caminiti, S. *et al.* (2007). On coding labeled trees. *Theoretical Computer Science*, **382**(2), 97–108. Latin American Theoretical Informatics.

Catanzaro, D. and Pesenti, R. (2019). Enumerating vertices of the balanced minimum evolution polytope. *Computers & Operations Research*, **109**, 209–217.

Neville, E. H. (1953). The codifying of tree-structure. *Mathematical Proceedings of the Cambridge Philosophical Society*, **49**(3), 381–385.

Prüfer, H. (1918). Neuer beweis eines satzes über permutationen. *Archiv für Mathematik und Physik*, pages 742–744.

Rohlf, J. (1983). Numbering binary trees with labeled terminal vertices. *Bulletin of Mathematical Biology*, **45**, 40.
